# Maternal Pre-Pregnancy BMI and Intelligence Quotient (IQ) in 5-Year-Old Children: A Cohort Based Study

**DOI:** 10.1371/journal.pone.0094498

**Published:** 2014-04-11

**Authors:** Mette Bliddal, Jørn Olsen, Henrik Støvring, Hanne-Lise F. Eriksen, Ulrik S. Kesmodel, Thorkild I. A. Sørensen, Ellen A. Nøhr

**Affiliations:** 1 Department of Gynaecology and Obstetrics, Odense University Hospital, and Institute of Clinical Research, Research Unit of Gynaecology and Obstetrics, University of Southern Denmark, Odense, Denmark; 2 Department of Public Health, Section for Epidemiology, Aarhus University, Aarhus, Denmark; 3 Department of Public Health, Section for Biostatistics, Aarhus University, Aarhus, Denmark; 4 Center of Functionally Integrative Neuroscience, Institute for Clinical Medicine, Aarhus University, Aarhus, Denmark; 5 Department of Obstetrics and Gynaecology, Aarhus University Hospital, Aarhus, Denmark; 6 Novo Nordisk Foundation Center for Basic Metabolic Research, University of Copenhagen, Denmark; 7 Institute of Preventive Medicine, Bispebjerg and Frederiksberg Hospitals – Part of Copenhagen University Hospital, Copenhagen, Denmark; Hamamatsu University School of Medicine, Japan

## Abstract

**Background:**

An association between maternal pre-pregnancy BMI and childhood intelligence quotient (IQ) has repeatedly been found but it is unknown if this association is causal or due to confounding caused by genetic or social factors.

**Methods:**

We used a cohort of 1,783 mothers and their 5-year-old children sampled from the Danish National Birth Cohort. The children participated between 2003 and 2008 in a neuropsychological assessment of cognitive ability including IQ tests taken by both the mother and the child. Linear regression analyses were used to estimate the associations between parental BMI and child IQ adjusted for a comprehensive set of potential confounders. Child IQ was assessed with the Wechsler Primary and Preschool Scales of Intelligence – Revised (WPPSI-R).

**Results:**

The crude association between maternal BMI and child IQ showed that BMI was adversely associated with child IQ with a reduction in IQ of −0.40 point for each one unit increase in BMI. This association was attenuated after adjustment for social factors and maternal IQ to a value of −0.27 (−0.50 to −0.03). After mutual adjustment for the father's BMI and all other factors except maternal IQ, the association between paternal BMI and child IQ yielded a regression coefficient of −0.26 (−0.59 to 0.07), which was comparable to that seen for maternal BMI (−0.20 (−0.44 to 0.04)).

**Conclusion:**

Although maternal pre-pregnancy BMI was inversely associated with the IQ of her child, the similar association with paternal BMI suggests that it is not a specific pregnancy related adiposity effect.

## Introduction

Maternal pre-pregnancy obesity is associated with numerous neonatal adverse outcomes such as increased risk of fetal death, macrosomia, and birth complications [1], [2]. Additionally, maternal obesity and excessive weight gain in pregnancy predict negative long-term physical health outcomes in the child such as obesity [3] and elevated blood pressure [4]. Fetal programming by factors induced by maternal obesity has been suggested [3].

If maternal obesity affects the fetal brain development through modifications of the intrauterine environment is not known but some studies have shown associations between maternal pre-pregnancy obesity and ADHD [5], inattention [6], neurodevelopmental disorders [7], and intellectual disability in children [8].

High BMI has been associated with a low IQ in adults and children [9]–[13] and childhood IQ, as a measure of general cognitive ability, has also been inversely associated with maternal pre-pregnancy BMI in some studies but not all [3], [14]–[16]. A recent study, investigating predictors for childhood IQ, found maternal BMI to be negatively associated with child IQ – apparently independent of maternal IQ - and hence maternal BMI was a predictor of child IQ [17].

If fat tissue is causally related to cognitive skills, causation may operate via the hormonal activities of fat tissue, by release of environmental fat-soluble toxins, or by an unbalanced diet leading to obesity. Some findings however, indicate that the association between maternal pregnancy BMI and child cognitive outcomes is due to confounding by genetic factors or post-natal social confounding [18]. As stated by Brion [19], it is important, given the global epidemic of obesity, to disentangle the biological effects of intrauterine exposure to maternal obesity on child cognitive development from spurious associations due to confounding. The degree of confounding can be assessed by a comparison of the associations between the maternal exposure and the child outcome with the association between paternal exposure and child IQ. If exposure to intrauterine excessive fat tissue is causally related to child IQ, one would expect the maternal association to be larger than the paternal association [19], [20]. Comparison of the maternal and paternal BMI association on child cognitive development as an estimate test of causality has only been done a few times [18], [21]. We add to this research more detailed data and estimate the association between maternal pre-pregnancy BMI, paternal BMI, and child IQ at the age of five taking into account potential confounding factors including maternal IQ.

Our hypothesis was that an association between maternal BMI and child IQ is not driven by a specific pregnancy programming effect related to fat tissue but rather by genetic and family confounders. We therefore expected to find not only an association for maternal BMI and child IQ but also for paternal BMI. If the association is only seen for maternal BMI, our hypothesis is not corroborated.

## Subjects and Methods

### Sample

This study was based on data from the ‘Lifestyle During Pregnancy Study (LDPS)’ nested within The Danish National Birth Cohort (DNBC). The DNBC is a cohort of approximately 92,000 pregnant women recruited from 1996 to 2002 at their first antenatal visit [22]. LDPS is a follow-up study focusing on prenatal lifestyle factors (primarily maternal alcohol exposure) and later neurodevelopment of the child [23]. A total of 3,478 women were invited to participate in LDPS between September 2003 and June 2008, when their children reached 60–64 months of age. The sampling was stratified on alcohol exposure with an oversampling of high exposures [23]. Of those invited, 1,783 children (51%) participated in an extensive three-hour assessment of cognitive ability and IQ tests were included for both the mother and the child. No substantial differences were seen between invited participants and non-participants in the LDPS [24]. The tests were carried out by 10 trained psychologists following standardised test procedures at test sites located in four cities in Denmark [23]. IQ of the child was assessed with the widely used Wechsler Primary and Preschool Scales of Intelligence – Revised (WPPSI-R) [25] assessing the IQ of children of 2–7 years of age.

Exclusion criteria were mother's or child's inability to speak Danish, impaired hearing or vision of the child to the extent that the test session could not be performed. Twins and children with congenital diseases prone to cause mental impairment (e.g. trisomy 21) were also excluded (n = 14).

### Exposure variables

The main exposure was maternal pre-pregnancy BMI. On the basis of self-reported information on weight and height from the first pregnancy interview in the DNBC, which took place at approximately 16^th^ weeks of gestation (inter-quartile range 13–19 weeks), maternal pre-pregnancy BMI (in kg/m^2^) was calculated. We had information on paternal BMI, provided by the mother, for 78% of the fathers (n = 1,376) from a postnatal interview 18 months after birth.

### Outcome variable

The WPPSI-R, used for estimating child IQ, comprises five verbal subtests and five performance subtests from which Verbal IQ (VIQ), Performance IQ (PIQ), and Full-Scale IQ's (FSIQ) are derived. The short form used in the present study included three verbal (arithmetic, information and vocabulary) and three performance subtests (block design, geometric design and object assembly). When at least two verbal and two performance subtests were available, the IQ's were prorated using standard procedures. At the time of the study, no Danish WPPSI-R norms were available, so Swedish norms were used to derive scores and IQs [26].

### Covariates

The measurement of maternal IQ was based on the mean of two verbal subtests (information and vocabulary) from the Wechsler Adult Intelligence Scale (WAIS) [27] and the Raven's Standard Progressive Matrices [28]. Raw scores of each test were standardised based on the results from the full sample and weighted equally in a combined score which was re-standardised to an IQ scale with a mean of 100 and a SD of 15.

A self-completed questionnaire was administered to the mothers with questions on maternal marital status, maternal and parental educational level (years in school plus years of post-school theoretical education), postnatal parental smoking in the home (yes/no), and duration of exclusive breastfeeding (months). The exact age of the child at the time of testing was categorised into 5 years and 1 month, 5 years and 2 months, 5 years and 3 months and 5 years and 4 months.

Birth weight (grams) and gestational age (days) were obtained from the Danish Medical Birth Registry. Maternal age as well as the sex and age of the child were obtained from The Danish Civil Registration System.

The following covariates were obtained from the prenatal telephone interview in the DNBC and coded as follows: parity (0, 1, 2+), maternal prenatal smoking (yes/no), and average alcohol intake per week during pregnancy (0, 1–4, 5–8, 9+).

### Statistical analyses

**Figure 1:**
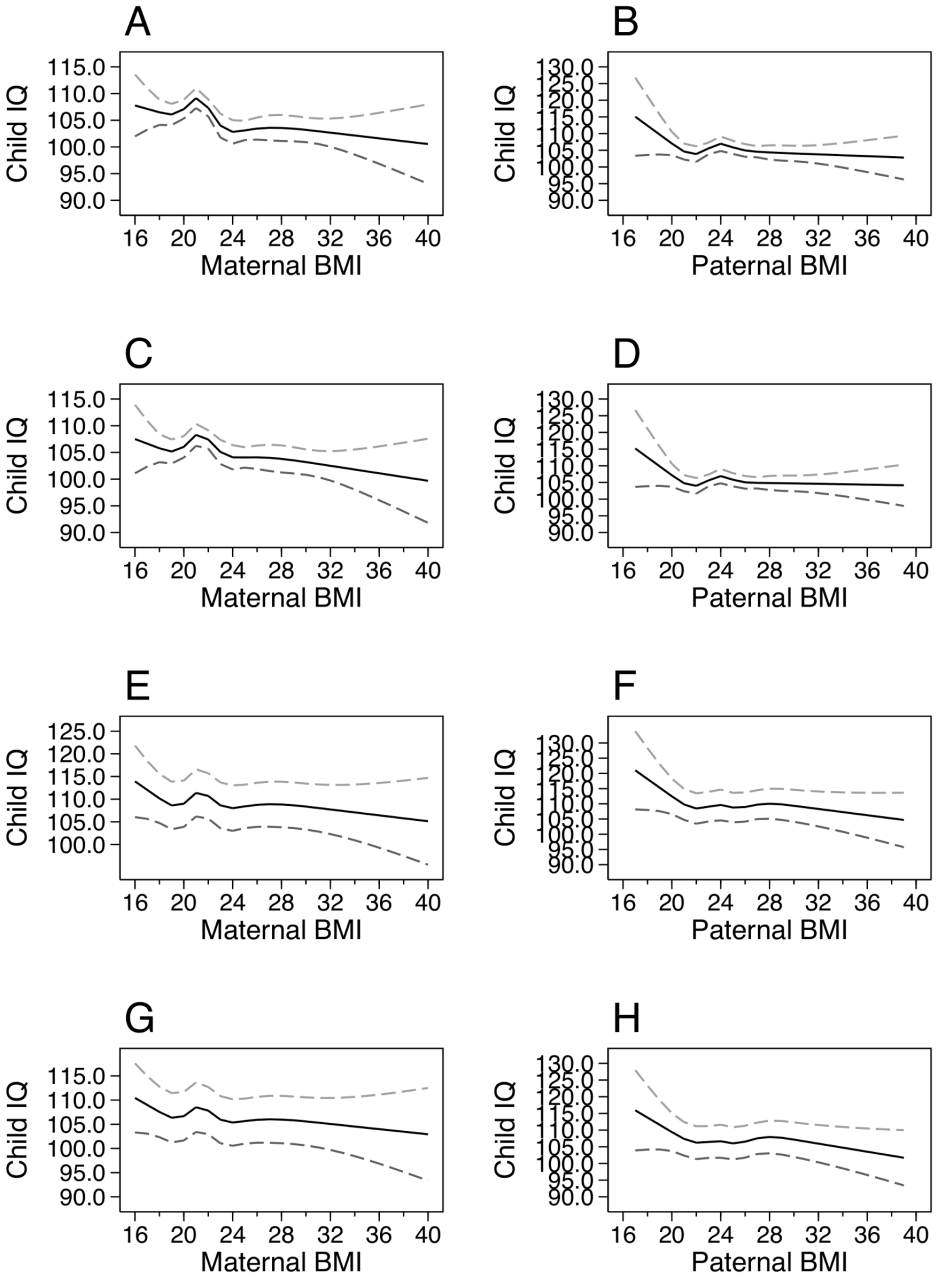
Splines for parental BMI and child IQ at the age of 5. Splines indicating estimates of child IQ according to maternal and paternal BMI. A. Maternal crude estimates. B. Paternal crude estimates. C. Maternal, mutually adjusted. D. Paternal, mutually adjusted. E. Maternal, adjusted for all covariates except maternal IQ. F. Paternal, adjusted for all covariates except maternal IQ. G. Maternal, adjusted for all covariates including maternal IQ. H. Paternal, adjusted for all covariates including maternal IQ.

Both maternal and paternal BMI distributions were approximately symmetric. Furthermore, all statistical analyses were weighted by sampling probabilities with robust variance estimation due to the selection method and oversampling of women with intake of alcohol during pregnancy [29]. This allows valid estimation of confidence intervals, even when the outcome variable is not normally distributed. Since a linear association between both maternal and paternal BMI and child IQ was compatible with the data (p = 0.46 and p = 0.30, respectively, based on a test for absence of a quadratic term in BMI) all analyses were done using linear regression on continuous BMI data. However we also generated restricted cubic splines (6 knots), allowing for a non-linear association to describe the associations between parental BMI and child IQ in more detail. The restricted cubic splines (Figure 1) displayed an inverse association between maternal BMI and child IQ in all the maternal analyses, however with a peak indicating highest IQ scores in children of women with a pre-pregnancy BMI of approximately 21. A similar inverse association was observed in restricted cubic splines of paternal BMI and child IQ with a peak at a paternal BMI at 24 units, but the deviation from a linear association was small.

In adjusted analyses, we included the other parent's BMI, gestational age, birth weight, sex of child, maternal age at birth, parity, marital status, maternal and paternal educational level, maternal prenatal smoking, postnatal smoking at home, average alcohol intake in pregnancy, breastfeeding and age of child when tested. Finally, maternal IQ was added to the model. We examined the correlation between maternal and paternal BMI and found a Pearson's correlations coefficient of 0.13.

We did the same analyses for maternal BMI and paternal BMI on verbal IQ and performance IQ to see if the results would differ on sub scales of IQ.

Supplementary analyses were stratified by sex of the child after excluding underweight women (n = 65).

Statistical estimates were two-sided with 95% confidence intervals. All statistical analyses were conducted in STATA 11 (StataCorp LP, College Station, TX, USA).

### Ethical approval

This study was approved by the Danish Data Protection Agency and access to the data was granted by the DNBC Steering Committee. According to Danish law, ethical approval is not required for registry-based studies.

## Results

Table 1 presents sample characteristics according to parental BMI. Of the 1,783 mothers included in the study, 7% were categorized as obese, 18% as overweight, 69% as normal weight, and 4% as underweight. The corresponding numbers in the smaller sample of fathers with data were 7%, 39%, 54% and 0.5%.

**Table 1:** Crude associations between parental BMI and selected covariates in the ‘Lifestyle during Pregnancy study.

				Maternal BMI				Paternal BMI	
				mean	(SD)			mean	(SD)
		n	%	Point		n	%	Point	
	All	1,783	100	23.47	(3.94)	1,376		25.10	(3.17)
**Maternal IQ**									
	<91	439	25	23.66	(3.99)	324	24	25.45	(3.14)
	91–100	435	25	23.61	(4.00)	338	25	25.08	(3.39)
	101–111	449	26	23.39	(3.96)	364	27	25.16	(3.06)
	>111	415	24	23.23	(3.83)	342	25	24.70	(3.08)
**Gestational age**									
	<32 weeks	3	0	22.25	(0.99)	2	0	25.43	(1.59)
	32–36 weeks	44	3	23.70	(5.69)	39	3	26.05	(3.21)
	37–41 weeks	1,540	88	23.42	(3.86)	1,211	88	25.00	(3.13)
	42+weeks	155	9	24.00	(4.28)	118	9	25.78	(3.51)
**Birth weight**									
	<2500 g	26	1	22.54	(4.69)	22	2	25.70	(3.07)
	25–3499 g	676	39	23.14	(3.93)	514	38	24.88	(2.98)
	35–4499 g	959	55	23.57	(3.81)	766	56	25.13	(3.12)
	4500+g	75	4	25.69	(4.91)	64	5	26.62	(4.67)
**Gender of child**									
	Girls	842	48	23.55	(3.95)	661	48	25.14	(3.08)
	Boys	904	52	23.40	(3.94)	714	52	25.07	(3.25)
**Maternal age at birth**									
	<21 years	20	1	22.53	(3.56)	12	1	24.09	(2.73)
	21–34 years	1,428	82	23.61	(4.07)	1,114	81	25.07	(3.24)
	35–39 years	264	15	22.84	(3.00)	221	16	25.35	(2.78)
	40+years	35	2	23.20	(4.60)	29	2	24.75	(3.62)
**Parity**									
	1	886	51	23.29	(3.74)	683	50	24.85	(2.98)
	2	562	32	23.70	(4.39)	457	33	25.30	(3.39)
	3+	299	17	23.59	(3.63)	236	17	25.44	(3.23)
**Marital status**									
	Cohabitant/married	1,517	88	23.47	(3.91)	1,231	90	25.14	(3.18)
	Single	215	12	23.52	(3.99)	135	10	24.68	(3.06)
**Maternal education index**									
	no additional education	192	11	23.65	(4.31)	151	11	25.43	(3.97)
	Special worker	20	1	23.17	(3.85)	10	1	25.17	(1.93)
	Other technical education	333	19	24.19	(4.32)	264	19	25.69	(3.40)
	Short academic education	323	19	23.65	(3.84)	260	19	25.10	(3.25)
	3–4½ academic education	592	34	23.33	(3.75)	465	34	24.95	(2.90)
	Long academic education	281	16	22.62	(3.36)	221	16	24.45	(2.63)
**Paternal education index**									
	no additional education	243	14	23.82	(3.73)	179	13	25.19	(3.77)
	Special worker	70	4	24.30	(3.74)	47	3	25.77	(2.94)
	Other technical education	523	30	24.39	(4.53)	436	32	25.43	(3.46)
	Short academic education	231	13	23.38	(3.76)	182	13	24.99	(2.73)
	3–4½ academic education	289	17	22.73	(3.31)	235	17	25.08	(2.99)
	Long academic education	351	20	22.30	(3.06)	274	20	24.51	(2.68)
	Not known	34	2	24.02	(4.91)	18	1	24.49	(2.70)
**Maternal smoking in pregnancy**									
	No	1,197	69	23.56	(4.00)	950	69	25.13	(3.19)
	Yes	549	31	23.29	(3.82)	425	31	25.05	(3.13)
**Post pregnancy smoking in the home**									
	No	1,179	68	23.47	(3.82)	947	69	25.09	(3.03)
	Yes	558	32	23.47	(3.95)	422	31	25.12	(3.49)
**Average alcohol intake in pregnancy**									
	No alcohol intake	836	48	23.68	(4.16)	661	48	25.02	(3.28)
	1–4 glass/week	714	41	23.29	(3.78)	555	40	25.12	(3.10)
	5–8 glass/week	177	10	23.36	(3.47)	142	10	25.34	(3.00)
	9+glass/week	19	1	22.64	(4.20)	17	1	25.76	(2.41)
**Solely breastfeeding**									
	Never solely breastfeed	50	3	25.01	(4.71)	36	3	25.15	(3.47)
	<4 full months	337	20	24.01	(4.31)	257	20	25.25	(3.49)
	4–6 months	1,141	69	23.26	(3.71)	904	70	24.99	(2.95)
	7+months	126	8	22.61	(3.23)	104	8	25.03	(3.03)
**Paternal BMI**									
	<18.5	6	0	22.83	(1.76)				
	18.5–24.9	733	54	23.16	(3.74)				
	25–29.9	526	39	23.58	(3.93)				
	30+	92	7	25.43	(5.37)				

Percentages are column percentages. Missing omitted. Data was missing on maternal BMI for 36 mothers, on child IQ for 11 children. Only few values were missing for potential confounder (0–15) except breastfeeding (missing 96).

We found an inverse trend between both maternal and paternal BMI and maternal IQ and educational status. Maternal BMI was associated with higher birth weight and longer gestational age, which was not seen for paternal BMI. For all other covariates, no strong association with parental BMI was found.

We observed an inverse association between maternal pre-pregnancy BMI and child IQ with a decrease of 0.40 IQ point (−0.64 to −0.17) for every unit increase in BMI (Table 2). Adjusting for maternal IQ attenuated this association to −0.30 IQ points (−0.51 to −0.08).

**Table 2:** Child IQ (age 5) according to maternal BMI.

		n	Coef.	(95% CI)	s.e.	p
Maternal BMI, crude		1,737	−0.40	(−0.64; −0.17)	0.12	0.001
Maternal BMI*		1,735	−0.30	(−0.51; −0.08)	0.11	0.007
Maternal BMI**		1,623	−0.27	(−0.50; −0.03)	0.12	0.030

*adjusted for maternal IQ.

**adjusted for all chosen covariates and maternal IQ.

The crude association between paternal BMI and child IQ was −0.23 IQ points (−0.56 to 0.10) (Table 3). Mutual adjustment for both parents BMI led, for paternal BMI, to numerically smaller estimates and when all other factors, except maternal IQ, were added to the model, the associations showed a regression coefficient of −0.26 IQ points (−0.59 to 0.07) for paternal BMI and child IQ and −0.20 (−0.44 to 0.04) for maternal BMI.

**Table 3:** Child IQ (age 5) according to maternal and paternal BMI.

		n	Coef.	(95% CI)	s.e.	p
Crude						
	Maternal BMI	1,349	−0.35	(−0.60; −0.11)	0.13	0.005
	Paternal BMI	1,368	−0.23	(−0.56; 0.10)	0.17	0.171
mutually adjusted only*						
	Maternal BMI	1,349	−0.34	(−0.58; −0.09)	0.13	0.008
	Paternal BMI	1,349	−0.17	(−0.50; 0.16)	0.17	0.319
Adjusted**						
	Maternal BMI	1,260	−0.20	(−0.44; 0.04)	0.12	0.111
	Paternal BMI	1,260	−0.26	(−0.59; 0.07)	0.17	0.124

*Restricted to participants where we had information on paternal BMI.

**Mutually adjusted and adjusted for all other covariates except maternal IQ.

We did the same analyses on sub levels of IQ – verbal IQ and performance IQ in the children – and the results remained approximately the same (Supplementary tables S1 – S4). Supplementary analyses showed similar results for boys and girls. Excluding underweight women (n = 65) from the analyses did not change the results much (not shown).

## Discussion

As predicted, we found an inverse association between maternal pre-pregnancy BMI and child IQ which persisted in an attenuated form after adjustment for confounders. When comparing associations for both maternal and paternal BMI, effect estimates were similar suggesting that the association between maternal excessive fat tissue and child IQ is not related to programming induced by maternal fat tissue during pregnancy. The association is most likely confounded by genetic and pre- and post-natal family factors, which could explain why this association is only seen in some studies depending on the association between BMI and IQ in the population.

Only few studies have explored the association between maternal obesity and child cognitive development [5]–[7], [18], [21], [30]–[33] or, more specifically, child IQ [14], [15]. In a Finnish study [15] an association between maternal BMI and child IQ was only found in the most recent cohort. Brion et al [18], who examined parental BMI and child verbal- and nonverbal skills and behavioural problems in two cohorts, reported inconsistent evidence across cohorts and associations that were not robust to adjustments. Most of the studies support an inverse association between maternal obesity and various cognitive child outcomes [5]–[7], [14], [21], [30]–[33] but more studies have been requested [16] e.g. by comparing the estimates of maternal and paternal influence [19], [20]. A recent study found an association between maternal BMI and child IQ, suggesting maternal BMI to be a minor predictor for child IQ at the age of 5 [17].

Previous studies all adjusted for selected social factors, but only two of them compared maternal and paternal associations [18], [21]. One of these studies found an association between cognitive functions of the child and maternal pre-pregnancy BMI, but not paternal BMI [21] concluding that the maternal association was likely to be causal although the maternal and paternal associations had overlapping confidence intervals [19]. The other study did not find an association between paternal overweight and any child cognitive performance or behaviour tests [18].

Lifestyle and psychosocial conditions during and after pregnancy may confound the relation between maternal BMI and child IQ. Family income, parental education and breastfeeding tend to be strong indicators for cognitive abilities in childhood [34]. Inconsistency is found for birth weight, duration of birth, maternal age at child's birth, and maternal smoking and alcohol intake during pregnancy [16], [24], [29], [35]. Lack of intellectual stimulation and suboptimal home conditions may also affect mental development of the child. Incorporation of maternal-paternal comparisons in our analyses adds information to the adjustments for family factors.

Inclusion of maternal IQ data is a key strength in our study. If the association between maternal BMI and child IQ is solely due to maternal genetic confounding, we expected the correlation to be much weaker after adjusting for maternal IQ, which we did not see, but we observed a modest attenuation of the association indicating that the genetic marker of maternal IQ plays a role. The fact that the sub-cohort was originally designed to investigate maternal alcohol intake in pregnancy with an oversampling in the cohort of high alcohol intake [23] might reduce the power of the study but not its validity.

The data on maternal pre-pregnancy BMI relied on self-reported information at the first telephone interview in the DNBC at the 16^th^ week of gestation. This self-reported anthropometric information has been validated relative to weights observed in the antenatal care at approximately 9^th^ week of gestation for at sub-cohort from the DNBC of 5,033 women. Throughout the entire BMI scale, the BMI was under-estimated with a mean of 0.66 kg, with a slightly increased under-reporting with increasing BMI. Yet, the BMI categories derived from the two BMI estimates agreed in 91.4% of the cases [36]. The information on paternal weight was based on reported information from the mothers 18 months after delivery and is the best data available. Accuracy of informant reports on estimates of the height and weight of family members has been investigated concluding that this information is highly predictive of the actually weight and height [37].

Although maternal pre-pregnancy BMI is inversely associated with the IQ of her child, our findings suggest that this association is not mediated by excessive maternal fat tissue present during pregnancy. There may be many good reasons for reducing overweight before entering procreation but fear of a negative effect on the child's intellectual capacity need not be one of them.

## Supporting Information

Table S1 **Verbal child IQ according to maternal BMI.**
(DOCX)

Table S2 **Verbal child IQ according to maternal and paternal BMI.**
(DOCX)

Table S3 **Performance child IQ according to maternal BMI.**
(DOCX)

Table S4 **Performance child IQ according to maternal and paternal BMI.**
(DOCX)
