# Supplementary material for: Maternal Pre-Pregnancy BMI and Intelligence Quotient (IQ) in 5-Year-Old Children: A Cohort Based Study
Source: PLoS One. 2014 Apr 11;9(4):e94498. doi: 10.1371/journal.pone.0094498 (PMC3984139; doi:10.1371/journal.pone.0094498)
Supplement: Table S2 — Verbal child IQ according to maternal and paternal BMI. (DOCX) [file pone.0094498.s002.docx]

Table S2. Verbal child IQ (age 5) according to maternal and paternal BMI

|  |  | n | Coef. | (95% CI) |
| --- | --- | --- | --- | --- |
| Crude | |  |  |  |
|  | Maternal BMI | 1,351 | -0.38 | (-0.58; -0.19) |
|  | Paternal BMI | 1,370 | -0.33 | (-0.59; -0.07) |
|  |  |  |  |  |
| Mutually adjusted only* | | |  |  |
|  | Maternal BMI | 1,351 | -0.36 | (-0.55; -0.16) |
|  | Paternal BMI | 1,351 | -0.27 | (-0.53; -0.01) |
|  |  |  |  |  |
| Adjusted** | |  |  |  |
|  | Maternal BMI | 1,262 | -0.19 | (-0.40; 0.02) |
|  | Paternal BMI | 1,262 | -0.29 | (-0.56; -0.01) |

* Restricted to participants where we had information on paternal BMI.

** Mutually adjusted and adjusted for all other covariates expect maternal IQ.
