# Supplementary material for: Maternal Pre-Pregnancy BMI and Intelligence Quotient (IQ) in 5-Year-Old Children: A Cohort Based Study
Source: PLoS One. 2014 Apr 11;9(4):e94498. doi: 10.1371/journal.pone.0094498 (PMC3984139; doi:10.1371/journal.pone.0094498)
Supplement: Table S3 — Performance child IQ according to maternal BMI. (DOCX) [file pone.0094498.s003.docx]

Table S3. Performance child IQ (age 5) according to maternal BMI

|  |  | n | Coef. | (95% CI) |
| --- | --- | --- | --- | --- |
| Maternal BMI, crude | | 1,740 | -0.30 | (-0.60; 0.00) |
| Maternal BMI* | | 1,736 | -0.19 | (-0.47; 0.08) |
| Maternal BMI** | | 1,622 | -0.23 | (-0.51; 0.05) |

*adjusted for maternal IQ

**adjusted for all chosen covariates and maternal IQ
