# Supplementary material for: Maternal Pre-Pregnancy BMI and Intelligence Quotient (IQ) in 5-Year-Old Children: A Cohort Based Study
Source: PLoS One. 2014 Apr 11;9(4):e94498. doi: 10.1371/journal.pone.0094498 (PMC3984139; doi:10.1371/journal.pone.0094498)
Supplement: Table S4 — Performance child IQ according to maternal and paternal BMI. (DOCX) [file pone.0094498.s004.docx]

Table S4. Performance child IQ (age 5) according to maternal and paternal BMI

|  |  | n | Coef. | (95% CI) |
| --- | --- | --- | --- | --- |
| Crude | |  |  |  |
|  | Maternal BMI | 1,351 | -0.22 | (-0.54; 0.10) |
|  | Paternal BMI | 1,370 | -0.09 | (-0.49; 0.31) |
|  |  |  |  |  |
| Mutually adjusted only* | | |  |  |
|  | Maternal BMI | 1,351 | -0.21 | (-0.53; 0.10) |
|  | Paternal BMI | 1,351 | -0.04 | (-0.45; 0.37) |
|  |  |  |  |  |
| Adjusted** | |  |  |  |
|  | Maternal BMI | 1,262 | -0.13 | (-0.44; 0.18) |
|  | Paternal BMI | 1,262 | -0.18 | (-0.61; 0.24) |

* Restricted to participants where we had information on paternal BMI.

** Mutually adjusted and adjusted for all other covariates expect maternal IQ.
